# *rac*-Dichlorido(1-{(diphenyl­phosphan­yl)[2-(diphenyl­phosphan­yl)phen­yl]meth­yl}ferrocene-κ^2^ *P*,*P*′)palladium(II) dimethyl sulfoxide disolvate

**DOI:** 10.1107/S160053681103618X

**Published:** 2011-09-14

**Authors:** Raffael Schuecker, Walter Weissensteiner, Kurt Mereiter

**Affiliations:** a Institute of Organic Chemistry, University of Vienna, Währingerstrasse 38, A-1090 Vienna, Austria; b Institute of Chemical Technologies and Analytics, Vienna University of Technology, Getreidemarkt 9/164SC, A-1060 Vienna, Austria

## Abstract

The racemic title compound, [FePdCl_2_(C_5_H_5_)(C_36_H_29_P_2_)]·2(CH_3_)_2_SO, features a Pd-chelating 1,3-diphosphine, which is substituted at a P-bearing asymmetric C atom by a ferrocenyl group. The Pd^II^ atom is in a distorted quadratic coordination by two P and two Cl atoms with bond lengths of 2.2414 (3) and 2.2438 (3) Å for Pd—P, and 2.3452 (3) and 2.3565 (3) Å for Pd—Cl. The conformation of the Pd complex is controlled by an intra­molecular slipped π–π stacking inter­action between a phenyl and a cyclo­penta­dienyl ring with corresponding C⋯C distances starting at 3.300 (2) Å and the distance between ring centroids being 3.674 (2) Å. The crystal structure is stabilized by C—H⋯Cl and C—H⋯O hydrogen bonds. The (CH_3_)_2_SO solvent mol­ecules are arranged in layers parallel to (101) and are linked in pairs by C—H⋯O inter­actions. One (CH_3_)_2_SO mol­ecule is orientationally disordered [occupancy ratio 0.8766 (17):0.1234 (17)] with sulfur in two positions at both sides of its C_2_O triangle.

## Related literature

For general information on ferrocene-based diphosphines and their applications in asymmetric catalysis, see: Togni (1996 ▶); Blaser *et al.* (2007 ▶); Dai & Hou (2010 ▶). For the synthesis, coordination behavior, and use in asymmetric catalysis of ligands based on [diphenyl­phosphanyl-(2-diphenyl­phos­phan­yl­phen­yl)meth­yl]-ferrocene, see: Schuecker *et al.* (2010 ▶); Lotz *et al.* (2010 ▶).
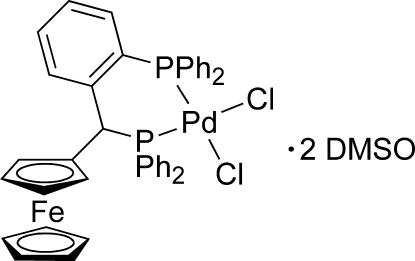


## Experimental

### 

#### Crystal data

[FePdCl_2_(C_5_H_5_)(C_36_H_29_P_2_)]·2C_2_H_6_OS
*M* *_r_* = 978.03
Triclinic, 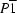

*a* = 10.9878 (8) Å
*b* = 11.5275 (8) Å
*c* = 17.1405 (12) Å
α = 78.720 (2)°
β = 81.796 (2)°
γ = 78.143 (2)°
*V* = 2071.8 (3) Å^3^
*Z* = 2
Mo *K*α radiation
μ = 1.13 mm^−1^
*T* = 100 K
0.59 × 0.45 × 0.36 mm

#### Data collection

Bruker SMART APEX CCD diffractometer
Absorption correction: multi-scan (*SADABS*; Bruker, 2003 ▶) *T*_min_ = 0.58, *T*_max_ = 0.67
37987 measured reflections
11991 independent reflections
11375 reflections with *I* > 2σ(*I*)
*R*_int_ = 0.018

#### Refinement

*R*[*F* ^2^ > 2σ(*F* ^2^)] = 0.021
*wR*(*F* ^2^) = 0.055
*S* = 1.02
11991 reflections
502 parameters
H-atom parameters constrained
Δρ_max_ = 0.59 e Å^−3^
Δρ_min_ = −0.60 e Å^−3^

### 

Data collection: *SMART* (Bruker, 2003 ▶); cell refinement: *SAINT* (Bruker, 2003 ▶); data reduction: *SAINT* and *XPREP* (Bruker, 2003 ▶); program(s) used to solve structure: *SHELXS97* (Sheldrick, 2008 ▶); program(s) used to refine structure: *SHELXL97* (Sheldrick, 2008 ▶); molecular graphics: *Mercury* (Macrae *et al.*, 2006 ▶); software used to prepare material for publication: *PLATON* (Spek, 2009 ▶) and *publCIF* (Westrip, 2010 ▶).

## Supplementary Material

Crystal structure: contains datablock(s) I, global. DOI: 10.1107/S160053681103618X/gk2404sup1.cif

Structure factors: contains datablock(s) I. DOI: 10.1107/S160053681103618X/gk2404Isup2.hkl

Additional supplementary materials:  crystallographic information; 3D view; checkCIF report

## Figures and Tables

**Table 1:** Hydrogen-bond geometry (Å, °)

*D*—H⋯*A*	*D*—H	H⋯*A*	*D*⋯*A*	*D*—H⋯*A*
C6—H6⋯O1^i^	1.00	2.40	3.3365 (17)	156
C15—H15⋯Cl1^ii^	0.95	2.70	3.4262 (12)	133
C19—H19⋯Cl1	0.95	2.66	3.2410 (12)	120
C23—H23⋯O1^i^	0.95	2.47	3.3239 (16)	149
C25—H25⋯Cl1	0.95	2.70	3.5075 (13)	143
C29—H29⋯O1^i^	0.95	2.45	3.3201 (16)	152
C31—H31⋯Cl2	0.95	2.76	3.3893 (13)	125
C42—H42*A*⋯O2	0.98	2.42	3.368 (2)	163
C45—H45*A*⋯Cl1^iii^	0.98	2.83	3.6881 (16)	147

Symmetry codes: (i) 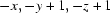; (ii) 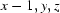; (iii) 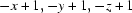.

**Fig. 1.**
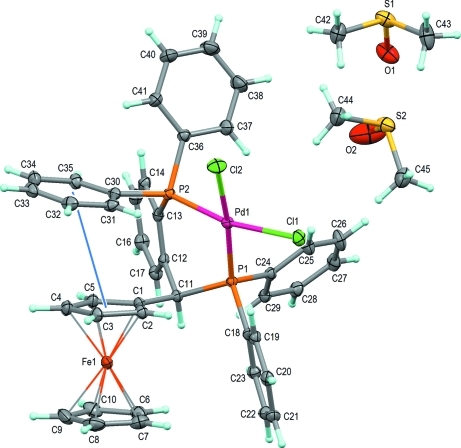
The asymmetric unit of (I) with displacement ellipsoids for the non-hydrogen atoms drawn at the 50% probability level. The blue line on the left links the centroids of ring C1—C5 and ring C30—C35, Cg···Cg = 3.674 (2) Å, to emphasize their π-π-stacking interaction with C3···C31 = 3.300 (2) Å as the shortest corresponding distance.

**Fig. 2.**
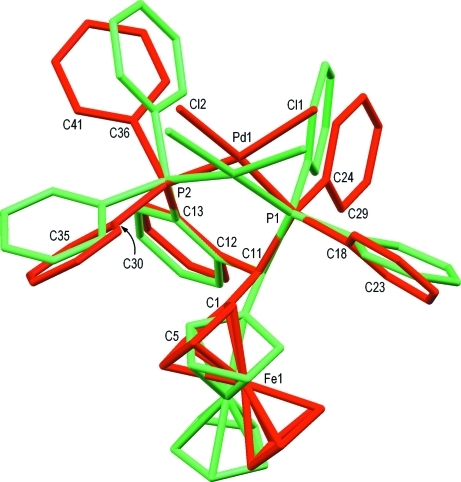
Comparison of the chemically identical Pd-complexes in the title compound, (I), red, and in non-racemic chloroform trisolvate (II), green, after least-squares fit of their P1—C11—C12—C13—P2 fragments (r.m.s. deviation 0.099 Å). Further positional differences (red-green) between the two molecules are: Pd1 0.55 Å, Cl1 1.38 Å, Cl2 0.70 Å, Fe1 1.18 Å, C1 0.49 Å, C18 0.36 Å, C24 0.69 Å, C29 1.12 Å, C30 0.61 Å, C35 1.45 Å, C36 0.58 Å, C41 1.41 Å. H-atoms omitted for clarity.

**Fig. 3.**
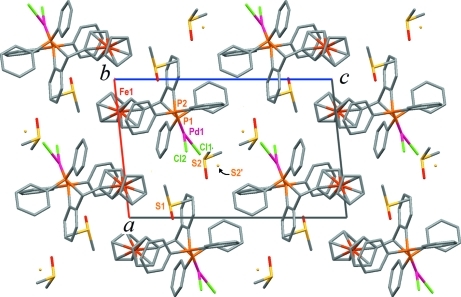
Packing diagram of (I) in a view along the b-axis showing the layer-like arrangement of the DMSO solvent molecules parallel to (101). S2' in this diagramm shows the location of the sulfur of the minor part of the orientation disordered 2nd DMSO molecule (C and O atoms identical for major and minor orienation). H-atoms omitted for clarity.

## supplementary crystallographic information

### Comment

Chiral ferrocenyldiphosphines are widely used as ligands for enantioselective PGM catalysts (PGM = platinum group metals) because they give excellent results in asymmetric hydrogenations and other transformations (Togni, 1996; Blaser *et al.*, 2007; Dai & Hou, 2010). Most of these ferrocenyldiphosphines are based on a planar chiral 1,2-disubstituted ferrocene backbone. Recently, it was found that chiral diphosphines lacking this planar-chiral backbone but employing ferrocene merely as a bulky substituent of an asymmetric *sp*^3^-carbon atom within a ferrocene-free 1,3-diphosphine system are also showing promising enantioselectivities for their PGM catalyst complexes (Schuecker *et al.*, 2010; Lotz *et al.*, 2010). Within the frame of a corresponding project the racemic title compound, (I), was synthesized and studied by X-ray diffraction before turning to the synthesis of the enantiopure equivalent recently reported by Lotz *et al.* (2010).

(I) is a dimethylsulfoxide (DMSO) solvate of the PdCl_2_ complex of racemic 2-[diphenylphosphanyl-(2-diphenylphosphanyl-phenyl)-methyl]-ferrocene and crystallizes in the centrosymmetric space group *P*1 with a cell content of two formula units. The asymmetric unit including the two DMSO molecules is shown in Fig. 1. Palladium has a distorted square planar coordination by two Cl atoms in terminal positions and by the two P atoms of the chelating 1,3-diphosphine ligand. The r.m.s. deviation from planarity of the PdCl_2_P_2_ fragment is 0.191 Å. Bond lengths in the Pd-complex of (I) show normal values and agree very well with those found in the crystal structure of its enantiopure chiral equivalent (Lotz *et al.*, 2010), which crystallizes as a chloroform trisolvate in the non-centrosymmetric space group *P*2_1_ and will be subsequently desgnated as (II). Disregarding a librating cyclopentadienyl ring in (II), the bond lengths in the Pd-complexes of (I) and (II) agree on the average within 0.006 Å with a maximum difference of 0.024 Å. With respect to bond angles and torsion angles the Pd-complexes in (I) and (II) show larger differences. Excluding ring bond angles the mean difference in bond angles is 2.3° with maximum differences of 7.6° for C24—P1—Pd1 and 5.6° for C18—P1—Pd1. The differences in torsion angles between (I) and (II) are even larger and lead to significantly differing conformations of both Pd-complexes, as demonstrated by Fig. 2. As result there is in (I) a distinct intramolecular π-π-stacking interaction between the phenyl ring C30—C35 and the monosubstituted cyclopentadienyl ring C1—C5 of the ferrocenyl moiety, which is entirely absent in the solid state structure of (II). Moreover are the phenyl rings of both complexes showing notably different orientations (Fig. 2). The intramolecular π-π-stacking interaction in (I) is characterized by C···C distances starting with 3.300 (2) Å (C3···C31) and a distance between ring centroids of 3.674 (2) Å (Fig. 1). The dihedral angle between the rings C1—C5 and C30—C35 is 10.85 (7)°. The torsion angles P1—C11—C1—C5 and C13—P2—C30—C35 are 160.05 (9)° and -38.11 (11)° in (I), and 104.8 (3)° and -71.1 (3)° in (II) (Lotz *et al.*, 2010), respectively.

A packing diagram of the structure of (I) is depicted in Fig. 3. The structure is stabilized by three intramolecular and two intermolecular C—H···Cl and four intermolecular C—H···O interactions listed in Table 1. The DMSO solvent molecules are arranged in layers parallel to (101). Their mutual interaction is limited to one C42—H···O2 bond.

### Experimental

A solution of racemic [diphenylphosphanyl-(2-diphenylphosphanylphenyl)methyl]-ferrocene (65 mg, 100 µmol; for synthesis, see: Lotz *et al.*, 2010) in benzene (2 ml) was added to a suspension of dichlorobis(acetonitrile) palladium(II) (26 mg, 100 µmol) in benzene (1 ml) and the resulting mixture was stirred at r.t. for 16 h. After filtration, the beige precipitate was washed with benzene and diethyl ether and was dried *in vacuo*. Crystals suitable for X-ray diffraction were obtained from a warm saturated solution of the complex in dimethylsulfoxide upon cooling to room temperature.

### Refinement

All H atoms were placed in calculated positions and thereafter treated as riding, C—H = 0.95 – 1.00 Å. *U*_ĩso_(H) = 1.2*U*_eq_(C) for CH groups; *U*_ĩso_(H) = 1.5*U*_eq_(C) for CH_3_ groups. A torsional parameter was refined for the methyl groups of the first dimethylsulfoxide molecule. Due to disorder the sulfur atom of the second dimethylsulfoxide molecule was found in two positions, S2 and S2' at both sides of its C44—C45—O2 triangle with site occupancies of 0.877 (2) and 0.123 (2). This disorder was modelled by two DMSO molecules having C44, C45, O2 in common, whereas the methyl hydrogen atoms of C44 and C45 were calculated either with S2 or S2'.

### Crystal data

**Table:**

[FePdCl_2_(C_5_H_5_)(C_36_H_29_P_2_)]·2C_2_H_6_OS	*Z* = 2
*M**_r_* = 978.03	*F*(000) = 1000
Triclinic, *P*1	*D*_x_ = 1.568 Mg m^−^^3^
*a* = 10.9878 (8) Å	Mo *K*α radiation, λ = 0.71073 Å
*b* = 11.5275 (8) Å	Cell parameters from 9252 reflections
*c* = 17.1405 (12) Å	θ = 2.4–30.0°
α = 78.720 (2)°	µ = 1.13 mm^−^^1^
β = 81.796 (2)°	*T* = 100 K
γ = 78.143 (2)°	Block, orange
*V* = 2071.8 (3) Å^3^	0.59 × 0.45 × 0.36 mm

### Data collection

**Table:**

Bruker SMART APEX CCD diffractometer	11991 independent reflections
Radiation source: fine-focus sealed tube	11375 reflections with *I* > 2σ(*I*)
graphite	*R*_int_ = 0.018
ω scans	θ_max_ = 30.0°, θ_min_ = 2.4°
Absorption correction: multi-scan (*SADABS*; Bruker, 2003)	*h* = −15→15
*T*_min_ = 0.58, *T*_max_ = 0.67	*k* = −16→16
37987 measured reflections	*l* = −24→24

### Refinement

**Table:**

Refinement on *F*^2^	Primary atom site location: structure-invariant direct methods
Least-squares matrix: full	Secondary atom site location: difference Fourier map
*R*[*F*^2^ > 2σ(*F*^2^)] = 0.021	Hydrogen site location: inferred from neighbouring sites
*wR*(*F*^2^) = 0.055	H-atom parameters constrained
*S* = 1.02	*w* = 1/[σ^2^(*F*_o_^2^) + (0.0275*P*)^2^ + 1.0316*P*] where *P* = (*F*_o_^2^ + 2*F*_c_^2^)/3
11991 reflections	(Δ/σ)_max_ = 0.002
502 parameters	Δρ_max_ = 0.59 e Å^−^^3^
0 restraints	Δρ_min_ = −0.60 e Å^−^^3^

### Special details

**Table:**

Geometry. All e.s.d.'s (except the e.s.d. in the dihedral angle between two l.s. planes) are estimated using the full covariance matrix. The cell e.s.d.'s are taken into account individually in the estimation of e.s.d.'s in distances, angles and torsion angles; correlations between e.s.d.'s in cell parameters are only used when they are defined by crystal symmetry. An approximate (isotropic) treatment of cell e.s.d.'s is used for estimating e.s.d.'s involving l.s. planes.
Refinement. Refinement of *F*^2^ against ALL reflections. The weighted *R*-factor *wR* and goodness of fit *S* are based on *F*^2^, conventional *R*-factors *R* are based on *F*, with *F* set to zero for negative *F*^2^. The threshold expression of *F*^2^ > σ(*F*^2^) is used only for calculating *R*-factors(gt) *etc*. and is not relevant to the choice of reflections for refinement. *R*-factors based on *F*^2^ are statistically about twice as large as those based on *F*, and *R*- factors based on ALL data will be even larger.

### Fractional atomic coordinates and isotropic or equivalent isotropic displacement parameters (Å^2^)

**Table:**

	*x*	*y*	*z*	*U*_iso_*/*U*_eq_	Occ. (<1)
Pd1	0.391661 (7)	0.309337 (7)	0.293169 (5)	0.01244 (3)	
Fe1	0.232918 (16)	0.590612 (15)	0.012021 (10)	0.01425 (4)	
Cl1	0.53877 (3)	0.36883 (3)	0.356324 (17)	0.01869 (5)	
Cl2	0.51837 (3)	0.11729 (3)	0.29591 (2)	0.02389 (6)	
P1	0.27906 (3)	0.49620 (2)	0.273579 (16)	0.01138 (5)	
P2	0.23545 (3)	0.23234 (3)	0.263806 (17)	0.01252 (5)	
C1	0.21553 (10)	0.48140 (10)	0.12186 (6)	0.01334 (19)	
C2	0.34258 (11)	0.46343 (10)	0.08620 (7)	0.0150 (2)	
H2	0.4127	0.4748	0.1085	0.018*	
C3	0.34579 (11)	0.42547 (11)	0.01137 (7)	0.0171 (2)	
H3	0.4221	0.4067	−0.0267	0.021*	
C4	0.22132 (12)	0.41994 (11)	0.00016 (7)	0.0186 (2)	
H4	0.1949	0.3965	−0.0472	0.022*	
C5	0.14085 (11)	0.45439 (11)	0.06794 (7)	0.0169 (2)	
H5	0.0481	0.4595	0.0763	0.020*	
C6	0.17661 (14)	0.76490 (11)	0.03013 (8)	0.0236 (3)	
H6	0.1486	0.7915	0.0830	0.028*	
C7	0.30156 (14)	0.74733 (12)	−0.00721 (9)	0.0263 (3)	
H7	0.3768	0.7595	0.0147	0.032*	
C8	0.30069 (16)	0.70912 (13)	−0.08132 (9)	0.0312 (3)	
H8	0.3753	0.6895	−0.1205	0.037*	
C9	0.17567 (17)	0.70275 (13)	−0.08939 (8)	0.0318 (3)	
H9	0.1480	0.6797	−0.1332	0.038*	
C10	0.09884 (14)	0.73710 (13)	−0.02031 (9)	0.0278 (3)	
H10	0.0064	0.7410	−0.0094	0.033*	
C11	0.16067 (10)	0.51763 (10)	0.20224 (6)	0.01247 (18)	
H11	0.1231	0.6052	0.1921	0.015*	
C12	0.05649 (10)	0.44928 (10)	0.23955 (6)	0.01310 (19)	
C13	0.07864 (10)	0.32422 (10)	0.26658 (7)	0.01338 (19)	
C14	−0.02352 (11)	0.26738 (11)	0.29802 (7)	0.0159 (2)	
H14	−0.0091	0.1831	0.3170	0.019*	
C15	−0.14507 (11)	0.33164 (11)	0.30205 (7)	0.0182 (2)	
H15	−0.2132	0.2914	0.3224	0.022*	
C16	−0.16641 (11)	0.45555 (11)	0.27610 (7)	0.0186 (2)	
H16	−0.2492	0.5005	0.2795	0.022*	
C17	−0.06631 (11)	0.51349 (11)	0.24516 (7)	0.0165 (2)	
H17	−0.0816	0.5981	0.2275	0.020*	
C18	0.37044 (10)	0.61313 (10)	0.22960 (6)	0.01320 (19)	
C19	0.49844 (11)	0.58615 (10)	0.20534 (7)	0.0152 (2)	
H19	0.5406	0.5048	0.2115	0.018*	
C20	0.56427 (11)	0.67840 (11)	0.17209 (7)	0.0177 (2)	
H20	0.6515	0.6599	0.1563	0.021*	
C21	0.50256 (12)	0.79743 (11)	0.16188 (7)	0.0186 (2)	
H21	0.5476	0.8603	0.1393	0.022*	
C22	0.37503 (12)	0.82434 (11)	0.18481 (7)	0.0192 (2)	
H22	0.3327	0.9056	0.1769	0.023*	
C23	0.30888 (11)	0.73305 (10)	0.21930 (7)	0.0166 (2)	
H23	0.2219	0.7522	0.2359	0.020*	
C24	0.19163 (10)	0.54361 (10)	0.36379 (7)	0.01424 (19)	
C25	0.24006 (12)	0.49419 (12)	0.43681 (7)	0.0208 (2)	
H25	0.3144	0.4351	0.4383	0.025*	
C26	0.17962 (13)	0.53136 (14)	0.50736 (8)	0.0252 (3)	
H26	0.2136	0.4988	0.5566	0.030*	
C27	0.06960 (12)	0.61611 (12)	0.50555 (7)	0.0215 (2)	
H27	0.0288	0.6420	0.5535	0.026*	
C28	0.01911 (12)	0.66312 (11)	0.43358 (7)	0.0188 (2)	
H28	−0.0575	0.7192	0.4328	0.023*	
C29	0.08018 (11)	0.62839 (10)	0.36261 (7)	0.0161 (2)	
H29	0.0463	0.6621	0.3134	0.019*	
C30	0.25815 (11)	0.17131 (10)	0.17153 (7)	0.0147 (2)	
C31	0.37585 (12)	0.15872 (10)	0.12720 (7)	0.0175 (2)	
H31	0.4430	0.1845	0.1442	0.021*	
C32	0.39411 (12)	0.10819 (11)	0.05810 (7)	0.0206 (2)	
H32	0.4736	0.1010	0.0274	0.025*	
C33	0.29749 (13)	0.06830 (11)	0.03384 (7)	0.0211 (2)	
H33	0.3116	0.0316	−0.0125	0.025*	
C34	0.17970 (12)	0.08172 (11)	0.07699 (7)	0.0202 (2)	
H34	0.1133	0.0548	0.0600	0.024*	
C35	0.15956 (11)	0.13480 (11)	0.14520 (7)	0.0175 (2)	
H35	0.0786	0.1462	0.1739	0.021*	
C36	0.21476 (11)	0.10941 (10)	0.34618 (7)	0.0150 (2)	
C37	0.20314 (13)	0.13400 (12)	0.42372 (8)	0.0221 (2)	
H37	0.2096	0.2116	0.4318	0.027*	
C38	0.18220 (16)	0.04528 (14)	0.48908 (8)	0.0297 (3)	
H38	0.1743	0.0624	0.5418	0.036*	
C39	0.17279 (14)	−0.06868 (13)	0.47762 (9)	0.0285 (3)	
H39	0.1571	−0.1289	0.5224	0.034*	
C40	0.18626 (13)	−0.09398 (12)	0.40114 (9)	0.0249 (3)	
H40	0.1815	−0.1722	0.3934	0.030*	
C41	0.20692 (12)	−0.00531 (11)	0.33513 (8)	0.0200 (2)	
H41	0.2156	−0.0232	0.2826	0.024*	
S1	0.08549 (3)	0.05862 (3)	0.80570 (2)	0.02640 (7)	
O1	−0.00595 (10)	0.17498 (11)	0.80041 (7)	0.0341 (2)	
C42	0.13223 (15)	0.03229 (15)	0.70553 (9)	0.0303 (3)	
H42A	0.1643	0.1019	0.6733	0.045*	
H42B	0.0601	0.0201	0.6825	0.045*	
H42C	0.1979	−0.0396	0.7058	0.045*	
C43	0.23073 (15)	0.09373 (19)	0.82039 (10)	0.0391 (4)	
H43A	0.2219	0.1229	0.8713	0.059*	
H43B	0.2541	0.1562	0.7765	0.059*	
H43C	0.2958	0.0213	0.8215	0.059*	
S2	0.42389 (4)	0.21103 (4)	0.62599 (2)	0.02793 (12)	0.8766 (17)
O2	0.28718 (13)	0.25821 (14)	0.62440 (12)	0.0647 (5)	0.8766 (17)
C44	0.47199 (17)	0.12803 (14)	0.54576 (9)	0.0331 (3)	0.8766 (17)
H44A	0.4356	0.0549	0.5577	0.050*	0.8766 (17)
H44B	0.5634	0.1060	0.5393	0.050*	0.8766 (17)
H44C	0.4434	0.1776	0.4962	0.050*	0.8766 (17)
C45	0.50322 (15)	0.33240 (14)	0.58599 (10)	0.0319 (3)	0.8766 (17)
H45A	0.4874	0.3894	0.6235	0.048*	0.8766 (17)
H45B	0.4728	0.3733	0.5348	0.048*	0.8766 (17)
H45C	0.5933	0.3016	0.5776	0.048*	0.8766 (17)
S2'	0.3760 (3)	0.2667 (3)	0.56587 (17)	0.02793 (12)	0.12
O2'	0.28718 (13)	0.25821 (14)	0.62440 (12)	0.0647 (5)	0.12
C44'	0.47199 (17)	0.12803 (14)	0.54576 (9)	0.0331 (3)	0.12
H44D	0.4226	0.0637	0.5598	0.050*	0.1234 (17)
H44E	0.5430	0.1080	0.5777	0.050*	0.1234 (17)
H44F	0.5029	0.1364	0.4888	0.050*	0.1234 (17)
C45'	0.50322 (15)	0.33240 (14)	0.58599 (10)	0.0319 (3)	0.12
H45D	0.4712	0.3876	0.6241	0.048*	0.1234 (17)
H45E	0.5385	0.3767	0.5360	0.048*	0.1234 (17)
H45F	0.5684	0.2683	0.6087	0.048*	0.1234 (17)

### Atomic displacement parameters (Å^2^)

**Table:**

	*U*^11^	*U*^22^	*U*^33^	*U*^12^	*U*^13^	*U*^23^
Pd1	0.01130 (4)	0.01214 (4)	0.01363 (4)	−0.00192 (3)	−0.00133 (3)	−0.00182 (3)
Fe1	0.01697 (8)	0.01527 (8)	0.01054 (7)	−0.00299 (6)	−0.00161 (6)	−0.00210 (6)
Cl1	0.01414 (11)	0.02339 (13)	0.01954 (13)	−0.00457 (10)	−0.00433 (9)	−0.00308 (10)
Cl2	0.02092 (13)	0.01617 (13)	0.03140 (16)	0.00267 (10)	−0.00350 (11)	−0.00233 (11)
P1	0.01208 (12)	0.01217 (12)	0.01047 (12)	−0.00285 (9)	−0.00086 (9)	−0.00290 (9)
P2	0.01299 (12)	0.01205 (12)	0.01289 (12)	−0.00271 (9)	0.00021 (9)	−0.00369 (10)
C1	0.0161 (5)	0.0133 (5)	0.0109 (4)	−0.0036 (4)	−0.0009 (4)	−0.0023 (4)
C2	0.0157 (5)	0.0161 (5)	0.0131 (5)	−0.0020 (4)	−0.0013 (4)	−0.0036 (4)
C3	0.0205 (5)	0.0169 (5)	0.0136 (5)	−0.0022 (4)	0.0004 (4)	−0.0049 (4)
C4	0.0246 (6)	0.0203 (5)	0.0136 (5)	−0.0077 (4)	−0.0009 (4)	−0.0067 (4)
C5	0.0191 (5)	0.0199 (5)	0.0140 (5)	−0.0073 (4)	−0.0018 (4)	−0.0044 (4)
C6	0.0333 (7)	0.0153 (5)	0.0191 (6)	−0.0003 (5)	−0.0011 (5)	−0.0014 (4)
C7	0.0309 (7)	0.0171 (6)	0.0307 (7)	−0.0078 (5)	−0.0022 (5)	−0.0007 (5)
C8	0.0439 (8)	0.0208 (6)	0.0226 (6)	−0.0060 (6)	0.0107 (6)	0.0013 (5)
C9	0.0536 (9)	0.0246 (7)	0.0159 (6)	−0.0031 (6)	−0.0125 (6)	0.0013 (5)
C10	0.0272 (7)	0.0240 (6)	0.0275 (7)	0.0025 (5)	−0.0081 (5)	0.0030 (5)
C11	0.0134 (5)	0.0130 (4)	0.0113 (4)	−0.0026 (4)	−0.0011 (4)	−0.0026 (4)
C12	0.0136 (5)	0.0160 (5)	0.0111 (4)	−0.0038 (4)	−0.0009 (4)	−0.0047 (4)
C13	0.0129 (5)	0.0156 (5)	0.0124 (5)	−0.0027 (4)	−0.0002 (4)	−0.0049 (4)
C14	0.0158 (5)	0.0171 (5)	0.0162 (5)	−0.0056 (4)	0.0003 (4)	−0.0048 (4)
C15	0.0150 (5)	0.0242 (6)	0.0171 (5)	−0.0070 (4)	0.0004 (4)	−0.0058 (4)
C16	0.0126 (5)	0.0237 (6)	0.0195 (5)	−0.0018 (4)	−0.0013 (4)	−0.0057 (4)
C17	0.0153 (5)	0.0176 (5)	0.0164 (5)	−0.0015 (4)	−0.0023 (4)	−0.0038 (4)
C18	0.0158 (5)	0.0143 (5)	0.0107 (4)	−0.0048 (4)	−0.0014 (4)	−0.0030 (4)
C19	0.0161 (5)	0.0170 (5)	0.0135 (5)	−0.0040 (4)	−0.0007 (4)	−0.0048 (4)
C20	0.0171 (5)	0.0224 (6)	0.0148 (5)	−0.0070 (4)	0.0008 (4)	−0.0043 (4)
C21	0.0226 (6)	0.0189 (5)	0.0157 (5)	−0.0096 (4)	−0.0006 (4)	−0.0015 (4)
C22	0.0229 (6)	0.0151 (5)	0.0189 (5)	−0.0039 (4)	−0.0013 (4)	−0.0013 (4)
C23	0.0172 (5)	0.0162 (5)	0.0161 (5)	−0.0030 (4)	−0.0009 (4)	−0.0028 (4)
C24	0.0160 (5)	0.0163 (5)	0.0117 (5)	−0.0056 (4)	0.0004 (4)	−0.0040 (4)
C25	0.0179 (5)	0.0297 (6)	0.0144 (5)	−0.0016 (5)	−0.0025 (4)	−0.0048 (5)
C26	0.0232 (6)	0.0396 (8)	0.0132 (5)	−0.0037 (5)	−0.0021 (4)	−0.0076 (5)
C27	0.0232 (6)	0.0280 (6)	0.0153 (5)	−0.0074 (5)	0.0034 (4)	−0.0093 (5)
C28	0.0209 (5)	0.0182 (5)	0.0178 (5)	−0.0037 (4)	0.0023 (4)	−0.0069 (4)
C29	0.0193 (5)	0.0150 (5)	0.0140 (5)	−0.0031 (4)	−0.0005 (4)	−0.0037 (4)
C30	0.0185 (5)	0.0122 (5)	0.0126 (5)	−0.0015 (4)	0.0002 (4)	−0.0027 (4)
C31	0.0200 (5)	0.0139 (5)	0.0176 (5)	−0.0034 (4)	0.0025 (4)	−0.0034 (4)
C32	0.0254 (6)	0.0164 (5)	0.0173 (5)	−0.0022 (4)	0.0053 (4)	−0.0040 (4)
C33	0.0304 (6)	0.0179 (5)	0.0133 (5)	0.0012 (5)	−0.0021 (4)	−0.0046 (4)
C34	0.0242 (6)	0.0201 (5)	0.0169 (5)	0.0001 (4)	−0.0068 (4)	−0.0059 (4)
C35	0.0179 (5)	0.0182 (5)	0.0163 (5)	−0.0002 (4)	−0.0024 (4)	−0.0054 (4)
C36	0.0153 (5)	0.0146 (5)	0.0150 (5)	−0.0038 (4)	−0.0003 (4)	−0.0023 (4)
C37	0.0311 (7)	0.0193 (5)	0.0163 (5)	−0.0051 (5)	−0.0002 (5)	−0.0050 (4)
C38	0.0436 (8)	0.0285 (7)	0.0150 (6)	−0.0068 (6)	0.0017 (5)	−0.0019 (5)
C39	0.0346 (7)	0.0258 (6)	0.0221 (6)	−0.0098 (6)	0.0018 (5)	0.0040 (5)
C40	0.0305 (7)	0.0179 (6)	0.0274 (7)	−0.0099 (5)	−0.0037 (5)	0.0000 (5)
C41	0.0262 (6)	0.0164 (5)	0.0189 (5)	−0.0063 (4)	−0.0032 (4)	−0.0038 (4)
S1	0.02146 (14)	0.02863 (16)	0.02352 (15)	−0.00114 (12)	−0.00013 (12)	0.00370 (12)
O1	0.0257 (5)	0.0393 (6)	0.0277 (5)	0.0091 (4)	0.0011 (4)	−0.0013 (4)
C42	0.0300 (7)	0.0348 (7)	0.0313 (7)	−0.0087 (6)	−0.0048 (6)	−0.0139 (6)
C43	0.0272 (7)	0.0624 (11)	0.0313 (8)	−0.0003 (7)	−0.0110 (6)	−0.0192 (8)
S2	0.0338 (2)	0.02294 (19)	0.02234 (19)	−0.00013 (15)	0.00061 (15)	−0.00055 (14)
O2	0.0291 (7)	0.0431 (8)	0.1074 (14)	−0.0019 (6)	0.0145 (8)	0.0012 (8)
C44	0.0452 (9)	0.0317 (7)	0.0280 (7)	−0.0149 (7)	−0.0077 (6)	−0.0078 (6)
C45	0.0342 (8)	0.0314 (7)	0.0346 (8)	−0.0069 (6)	−0.0073 (6)	−0.0132 (6)
S2'	0.0338 (2)	0.02294 (19)	0.02234 (19)	−0.00013 (15)	0.00061 (15)	−0.00055 (14)
O2'	0.0291 (7)	0.0431 (8)	0.1074 (14)	−0.0019 (6)	0.0145 (8)	0.0012 (8)
C44'	0.0452 (9)	0.0317 (7)	0.0280 (7)	−0.0149 (7)	−0.0077 (6)	−0.0078 (6)
C45'	0.0342 (8)	0.0314 (7)	0.0346 (8)	−0.0069 (6)	−0.0073 (6)	−0.0132 (6)

### Geometric parameters (Å, °)

**Table:**

Pd1—P1	2.2414 (3)	C20—H20	0.9500
Pd1—P2	2.2438 (3)	C21—C22	1.3878 (17)
Pd1—Cl1	2.3452 (3)	C21—H21	0.9500
Pd1—Cl2	2.3565 (3)	C22—C23	1.3905 (16)
Fe1—C10	2.0429 (13)	C22—H22	0.9500
Fe1—C9	2.0463 (14)	C23—H23	0.9500
Fe1—C6	2.0479 (13)	C24—C25	1.4003 (16)
Fe1—C5	2.0481 (12)	C24—C29	1.4010 (16)
Fe1—C4	2.0482 (12)	C25—C26	1.3941 (17)
Fe1—C3	2.0485 (12)	C25—H25	0.9500
Fe1—C7	2.0487 (14)	C26—C27	1.3893 (19)
Fe1—C2	2.0494 (11)	C26—H26	0.9500
Fe1—C8	2.0502 (14)	C27—C28	1.3905 (18)
Fe1—C1	2.0579 (11)	C27—H27	0.9500
P1—C24	1.8175 (11)	C28—C29	1.3919 (16)
P1—C18	1.8265 (11)	C28—H28	0.9500
P1—C11	1.8580 (11)	C29—H29	0.9500
P2—C30	1.8192 (12)	C30—C35	1.3984 (17)
P2—C36	1.8197 (12)	C30—C31	1.3987 (16)
P2—C13	1.8273 (11)	C31—C32	1.3914 (17)
C1—C2	1.4327 (15)	C31—H31	0.9500
C1—C5	1.4347 (16)	C32—C33	1.3831 (19)
C1—C11	1.5233 (15)	C32—H32	0.9500
C2—C3	1.4277 (16)	C33—C34	1.3912 (18)
C2—H2	0.9500	C33—H33	0.9500
C3—C4	1.4237 (17)	C34—C35	1.3931 (16)
C3—H3	1.0000	C34—H34	0.9500
C4—C5	1.4255 (16)	C35—H35	0.9500
C4—H4	1.0000	C36—C41	1.3935 (16)
C5—H5	1.0000	C36—C37	1.3953 (17)
C6—C10	1.419 (2)	C37—C38	1.3889 (18)
C6—C7	1.422 (2)	C37—H37	0.9500
C6—H6	1.0000	C38—C39	1.392 (2)
C7—C8	1.425 (2)	C38—H38	0.9500
C7—H7	1.0000	C39—C40	1.379 (2)
C8—C9	1.418 (2)	C39—H39	0.9500
C8—H8	1.0000	C40—C41	1.3951 (18)
C9—C10	1.426 (2)	C40—H40	0.9500
C9—H9	0.9500	C41—H41	0.9500
C10—H10	1.0000	S1—O1	1.4971 (11)
C11—C12	1.5181 (15)	S1—C42	1.7867 (16)
C11—H11	1.0000	S1—C43	1.7872 (17)
C12—C17	1.3993 (15)	C42—H42A	0.9800
C12—C13	1.4068 (15)	C42—H42B	0.9800
C13—C14	1.4040 (15)	C42—H42C	0.9800
C14—C15	1.3873 (16)	C43—H43A	0.9800
C14—H14	0.9500	C43—H43B	0.9800
C15—C16	1.3916 (18)	C43—H43C	0.9800
C15—H15	0.9500	S2—O2	1.4909 (15)
C16—C17	1.3907 (17)	S2—C45	1.7727 (17)
C16—H16	0.9500	S2—C44	1.7849 (16)
C17—H17	0.9500	C44—H44A	0.9800
C18—C19	1.3970 (16)	C44—H44B	0.9800
C18—C23	1.3979 (16)	C44—H44C	0.9800
C19—C20	1.3930 (16)	C45—H45A	0.9800
C19—H19	0.9500	C45—H45B	0.9800
C20—C21	1.3894 (17)	C45—H45C	0.9800
			
P1—Pd1—P2	91.550 (12)	C1—C11—P1	113.39 (8)
P1—Pd1—Cl1	92.209 (11)	C12—C11—H11	107.6
P2—Pd1—Cl1	165.791 (11)	C1—C11—H11	107.6
P1—Pd1—Cl2	172.603 (11)	P1—C11—H11	107.6
P2—Pd1—Cl2	88.084 (12)	C17—C12—C13	119.19 (10)
Cl1—Pd1—Cl2	89.922 (12)	C17—C12—C11	118.27 (10)
C10—Fe1—C9	40.80 (6)	C13—C12—C11	122.52 (10)
C10—Fe1—C6	40.58 (6)	C14—C13—C12	118.88 (10)
C9—Fe1—C6	68.38 (6)	C14—C13—P2	118.37 (9)
C10—Fe1—C5	106.71 (6)	C12—C13—P2	122.75 (8)
C9—Fe1—C5	121.41 (6)	C15—C14—C13	121.43 (11)
C6—Fe1—C5	123.21 (5)	C15—C14—H14	119.3
C10—Fe1—C4	122.88 (6)	C13—C14—H14	119.3
C9—Fe1—C4	106.95 (6)	C14—C15—C16	119.48 (11)
C6—Fe1—C4	159.41 (6)	C14—C15—H15	120.3
C5—Fe1—C4	40.73 (5)	C16—C15—H15	120.3
C10—Fe1—C3	159.58 (6)	C17—C16—C15	119.89 (11)
C9—Fe1—C3	123.41 (6)	C17—C16—H16	120.1
C6—Fe1—C3	158.59 (5)	C15—C16—H16	120.1
C5—Fe1—C3	68.51 (5)	C16—C17—C12	121.12 (11)
C4—Fe1—C3	40.67 (5)	C16—C17—H17	119.4
C10—Fe1—C7	68.38 (6)	C12—C17—H17	119.4
C9—Fe1—C7	68.36 (6)	C19—C18—C23	119.52 (10)
C6—Fe1—C7	40.61 (6)	C19—C18—P1	122.13 (9)
C5—Fe1—C7	160.03 (5)	C23—C18—P1	118.35 (9)
C4—Fe1—C7	158.21 (6)	C20—C19—C18	120.09 (11)
C3—Fe1—C7	122.83 (5)	C20—C19—H19	120.0
C10—Fe1—C2	157.83 (5)	C18—C19—H19	120.0
C9—Fe1—C2	160.25 (6)	C21—C20—C19	120.14 (11)
C6—Fe1—C2	122.59 (5)	C21—C20—H20	119.9
C5—Fe1—C2	68.57 (5)	C19—C20—H20	119.9
C4—Fe1—C2	68.56 (5)	C22—C21—C20	119.88 (11)
C3—Fe1—C2	40.78 (4)	C22—C21—H21	120.1
C7—Fe1—C2	108.25 (5)	C20—C21—H21	120.1
C10—Fe1—C8	68.39 (6)	C21—C22—C23	120.42 (11)
C9—Fe1—C8	40.51 (7)	C21—C22—H22	119.8
C6—Fe1—C8	68.34 (6)	C23—C22—H22	119.8
C5—Fe1—C8	157.36 (6)	C22—C23—C18	119.94 (11)
C4—Fe1—C8	122.00 (6)	C22—C23—H23	120.0
C3—Fe1—C8	107.94 (5)	C18—C23—H23	120.0
C7—Fe1—C8	40.70 (6)	C25—C24—C29	119.37 (11)
C2—Fe1—C8	124.29 (6)	C25—C24—P1	117.94 (9)
C10—Fe1—C1	121.48 (5)	C29—C24—P1	122.68 (9)
C9—Fe1—C1	157.32 (6)	C26—C25—C24	120.27 (12)
C6—Fe1—C1	107.35 (5)	C26—C25—H25	119.9
C5—Fe1—C1	40.90 (4)	C24—C25—H25	119.9
C4—Fe1—C1	68.78 (5)	C27—C26—C25	119.95 (12)
C3—Fe1—C1	68.75 (5)	C27—C26—H26	120.0
C7—Fe1—C1	123.83 (5)	C25—C26—H26	120.0
C2—Fe1—C1	40.83 (4)	C26—C27—C28	120.10 (11)
C8—Fe1—C1	160.61 (6)	C26—C27—H27	120.0
C24—P1—C18	105.64 (5)	C28—C27—H27	120.0
C24—P1—C11	105.04 (5)	C27—C28—C29	120.33 (12)
C18—P1—C11	102.85 (5)	C27—C28—H28	119.8
C24—P1—Pd1	114.22 (4)	C29—C28—H28	119.8
C18—P1—Pd1	114.46 (4)	C28—C29—C24	119.94 (11)
C11—P1—Pd1	113.50 (4)	C28—C29—H29	120.0
C30—P2—C36	107.35 (5)	C24—C29—H29	120.0
C30—P2—C13	105.54 (5)	C35—C30—C31	119.63 (11)
C36—P2—C13	101.44 (5)	C35—C30—P2	120.61 (9)
C30—P2—Pd1	117.87 (4)	C31—C30—P2	119.75 (9)
C36—P2—Pd1	104.80 (4)	C32—C31—C30	119.66 (12)
C13—P2—Pd1	118.14 (4)	C32—C31—H31	120.2
C2—C1—C5	107.21 (10)	C30—C31—H31	120.2
C2—C1—C11	130.02 (10)	C33—C32—C31	120.50 (12)
C5—C1—C11	122.74 (10)	C33—C32—H32	119.7
C2—C1—Fe1	69.26 (6)	C31—C32—H32	119.7
C5—C1—Fe1	69.18 (6)	C32—C33—C34	120.25 (11)
C11—C1—Fe1	128.18 (8)	C32—C33—H33	119.9
C3—C2—C1	108.31 (10)	C34—C33—H33	119.9
C3—C2—Fe1	69.58 (7)	C33—C34—C35	119.71 (12)
C1—C2—Fe1	69.91 (6)	C33—C34—H34	120.1
C3—C2—H2	125.8	C35—C34—H34	120.1
C1—C2—H2	125.8	C34—C35—C30	120.18 (11)
Fe1—C2—H2	126.2	C34—C35—H35	119.9
C4—C3—C2	108.07 (10)	C30—C35—H35	119.9
C4—C3—Fe1	69.65 (7)	C41—C36—C37	119.36 (11)
C2—C3—Fe1	69.65 (7)	C41—C36—P2	123.21 (9)
C4—C3—H3	126.0	C37—C36—P2	117.41 (9)
C2—C3—H3	126.0	C38—C37—C36	120.17 (12)
Fe1—C3—H3	126.0	C38—C37—H37	119.9
C3—C4—C5	108.05 (10)	C36—C37—H37	119.9
C3—C4—Fe1	69.67 (7)	C37—C38—C39	120.20 (13)
C5—C4—Fe1	69.63 (7)	C37—C38—H38	119.9
C3—C4—H4	126.0	C39—C38—H38	119.9
C5—C4—H4	126.0	C40—C39—C38	119.83 (12)
Fe1—C4—H4	126.0	C40—C39—H39	120.1
C4—C5—C1	108.36 (10)	C38—C39—H39	120.1
C4—C5—Fe1	69.64 (7)	C39—C40—C41	120.36 (12)
C1—C5—Fe1	69.92 (6)	C39—C40—H40	119.8
C4—C5—H5	125.8	C41—C40—H40	119.8
C1—C5—H5	125.8	C36—C41—C40	120.07 (12)
Fe1—C5—H5	125.8	C36—C41—H41	120.0
C10—C6—C7	108.11 (12)	C40—C41—H41	120.0
C10—C6—Fe1	69.52 (8)	O1—S1—C42	106.70 (7)
C7—C6—Fe1	69.73 (8)	O1—S1—C43	105.72 (8)
C10—C6—H6	125.9	C42—S1—C43	95.67 (7)
C7—C6—H6	125.9	S1—C42—H42A	109.5
Fe1—C6—H6	125.9	S1—C42—H42B	109.5
C6—C7—C8	107.89 (13)	H42A—C42—H42B	109.5
C6—C7—Fe1	69.66 (8)	S1—C42—H42C	109.5
C8—C7—Fe1	69.71 (8)	H42A—C42—H42C	109.5
C6—C7—H7	126.1	H42B—C42—H42C	109.5
C8—C7—H7	126.1	S1—C43—H43A	109.5
Fe1—C7—H7	126.1	S1—C43—H43B	109.5
C9—C8—C7	108.01 (13)	H43A—C43—H43B	109.5
C9—C8—Fe1	69.60 (8)	S1—C43—H43C	109.5
C7—C8—Fe1	69.59 (8)	H43A—C43—H43C	109.5
C9—C8—H8	126.0	H43B—C43—H43C	109.5
C7—C8—H8	126.0	O2—S2—C45	107.64 (8)
Fe1—C8—H8	126.0	O2—S2—C44	106.84 (10)
C8—C9—C10	108.00 (13)	C45—S2—C44	97.72 (8)
C8—C9—Fe1	69.90 (8)	S2—C44—H44A	109.5
C10—C9—Fe1	69.47 (8)	S2—C44—H44B	109.5
C8—C9—H9	126.0	H44A—C44—H44B	109.5
C10—C9—H9	126.0	S2—C44—H44C	109.5
Fe1—C9—H9	126.2	H44A—C44—H44C	109.5
C6—C10—C9	107.99 (13)	H44B—C44—H44C	109.5
C6—C10—Fe1	69.90 (8)	S2—C45—H45A	109.5
C9—C10—Fe1	69.72 (8)	S2—C45—H45B	109.5
C6—C10—H10	126.0	H45A—C45—H45B	109.5
C9—C10—H10	126.0	S2—C45—H45C	109.5
Fe1—C10—H10	126.0	H45A—C45—H45C	109.5
C12—C11—C1	110.27 (9)	H45B—C45—H45C	109.5
C12—C11—P1	110.01 (7)		
			
P2—Pd1—P1—C24	93.37 (4)	C4—Fe1—C7—C8	44.01 (19)
Cl1—Pd1—P1—C24	−72.92 (4)	C3—Fe1—C7—C8	79.17 (10)
P2—Pd1—P1—C18	−144.67 (4)	C2—Fe1—C7—C8	121.76 (9)
Cl1—Pd1—P1—C18	49.04 (4)	C1—Fe1—C7—C8	164.19 (9)
P2—Pd1—P1—C11	−27.02 (4)	C6—C7—C8—C9	−0.22 (15)
Cl1—Pd1—P1—C11	166.69 (4)	Fe1—C7—C8—C9	59.19 (10)
P1—Pd1—P2—C30	111.23 (4)	C6—C7—C8—Fe1	−59.42 (9)
Cl1—Pd1—P2—C30	−143.47 (5)	C10—Fe1—C8—C9	−37.83 (9)
Cl2—Pd1—P2—C30	−61.37 (4)	C6—Fe1—C8—C9	−81.64 (9)
P1—Pd1—P2—C36	−129.51 (4)	C5—Fe1—C8—C9	43.75 (18)
Cl1—Pd1—P2—C36	−24.21 (6)	C4—Fe1—C8—C9	78.34 (10)
Cl2—Pd1—P2—C36	57.88 (4)	C3—Fe1—C8—C9	120.80 (9)
P1—Pd1—P2—C13	−17.55 (4)	C7—Fe1—C8—C9	−119.36 (13)
Cl1—Pd1—P2—C13	87.75 (6)	C2—Fe1—C8—C9	162.85 (8)
Cl2—Pd1—P2—C13	169.84 (4)	C1—Fe1—C8—C9	−162.33 (14)
C10—Fe1—C1—C2	−162.23 (8)	C10—Fe1—C8—C7	81.54 (9)
C9—Fe1—C1—C2	164.57 (13)	C9—Fe1—C8—C7	119.36 (13)
C6—Fe1—C1—C2	−120.11 (7)	C6—Fe1—C8—C7	37.73 (9)
C5—Fe1—C1—C2	118.84 (9)	C5—Fe1—C8—C7	163.11 (13)
C4—Fe1—C1—C2	81.34 (7)	C4—Fe1—C8—C7	−162.30 (8)
C3—Fe1—C1—C2	37.56 (7)	C3—Fe1—C8—C7	−119.83 (9)
C7—Fe1—C1—C2	−78.53 (8)	C2—Fe1—C8—C7	−77.79 (10)
C8—Fe1—C1—C2	−46.18 (18)	C1—Fe1—C8—C7	−43.0 (2)
C10—Fe1—C1—C5	78.93 (9)	C7—C8—C9—C10	0.04 (16)
C9—Fe1—C1—C5	45.73 (16)	Fe1—C8—C9—C10	59.23 (10)
C6—Fe1—C1—C5	121.05 (8)	C7—C8—C9—Fe1	−59.19 (10)
C4—Fe1—C1—C5	−37.50 (7)	C10—Fe1—C9—C8	119.24 (13)
C3—Fe1—C1—C5	−81.28 (7)	C6—Fe1—C9—C8	81.53 (9)
C7—Fe1—C1—C5	162.63 (8)	C5—Fe1—C9—C8	−161.83 (8)
C2—Fe1—C1—C5	−118.84 (9)	C4—Fe1—C9—C8	−119.74 (9)
C8—Fe1—C1—C5	−165.02 (15)	C3—Fe1—C9—C8	−78.23 (10)
C10—Fe1—C1—C11	−36.99 (12)	C7—Fe1—C9—C8	37.69 (9)
C9—Fe1—C1—C11	−70.19 (18)	C2—Fe1—C9—C8	−46.1 (2)
C6—Fe1—C1—C11	5.13 (11)	C1—Fe1—C9—C8	164.85 (12)
C5—Fe1—C1—C11	−115.92 (12)	C6—Fe1—C9—C10	−37.71 (9)
C4—Fe1—C1—C11	−153.42 (11)	C5—Fe1—C9—C10	78.93 (10)
C3—Fe1—C1—C11	162.80 (11)	C4—Fe1—C9—C10	121.02 (9)
C7—Fe1—C1—C11	46.71 (12)	C3—Fe1—C9—C10	162.53 (8)
C2—Fe1—C1—C11	125.24 (12)	C7—Fe1—C9—C10	−81.55 (10)
C8—Fe1—C1—C11	79.06 (19)	C2—Fe1—C9—C10	−165.36 (14)
C5—C1—C2—C3	−0.18 (13)	C8—Fe1—C9—C10	−119.24 (13)
C11—C1—C2—C3	177.79 (11)	C1—Fe1—C9—C10	45.61 (18)
Fe1—C1—C2—C3	−59.17 (8)	C7—C6—C10—C9	−0.30 (15)
C5—C1—C2—Fe1	59.00 (8)	Fe1—C6—C10—C9	−59.55 (10)
C11—C1—C2—Fe1	−123.04 (12)	C7—C6—C10—Fe1	59.25 (9)
C10—Fe1—C2—C3	163.17 (14)	C8—C9—C10—C6	0.16 (16)
C9—Fe1—C2—C3	−42.78 (19)	Fe1—C9—C10—C6	59.66 (9)
C6—Fe1—C2—C3	−161.90 (7)	C8—C9—C10—Fe1	−59.50 (10)
C5—Fe1—C2—C3	81.52 (7)	C9—Fe1—C10—C6	−119.06 (13)
C4—Fe1—C2—C3	37.61 (7)	C5—Fe1—C10—C6	121.92 (8)
C7—Fe1—C2—C3	−119.46 (8)	C4—Fe1—C10—C6	163.50 (8)
C8—Fe1—C2—C3	−77.31 (9)	C3—Fe1—C10—C6	−164.95 (13)
C1—Fe1—C2—C3	119.55 (10)	C7—Fe1—C10—C6	−37.57 (8)
C10—Fe1—C2—C1	43.62 (17)	C2—Fe1—C10—C6	47.85 (18)
C9—Fe1—C2—C1	−162.33 (16)	C8—Fe1—C10—C6	−81.50 (9)
C6—Fe1—C2—C1	78.55 (8)	C1—Fe1—C10—C6	79.79 (9)
C5—Fe1—C2—C1	−38.03 (6)	C6—Fe1—C10—C9	119.06 (13)
C4—Fe1—C2—C1	−81.94 (7)	C5—Fe1—C10—C9	−119.01 (9)
C3—Fe1—C2—C1	−119.55 (10)	C4—Fe1—C10—C9	−77.44 (10)
C7—Fe1—C2—C1	120.99 (7)	C3—Fe1—C10—C9	−45.9 (2)
C8—Fe1—C2—C1	163.14 (7)	C7—Fe1—C10—C9	81.50 (10)
C1—C2—C3—C4	0.11 (13)	C2—Fe1—C10—C9	166.92 (13)
Fe1—C2—C3—C4	−59.26 (8)	C8—Fe1—C10—C9	37.56 (10)
C1—C2—C3—Fe1	59.38 (8)	C1—Fe1—C10—C9	−161.15 (9)
C10—Fe1—C3—C4	−42.40 (18)	C2—C1—C11—C12	−141.51 (12)
C9—Fe1—C3—C4	−76.60 (9)	C5—C1—C11—C12	36.18 (14)
C6—Fe1—C3—C4	165.15 (13)	Fe1—C1—C11—C12	124.36 (9)
C5—Fe1—C3—C4	37.68 (7)	C2—C1—C11—P1	−17.64 (15)
C7—Fe1—C3—C4	−160.86 (8)	C5—C1—C11—P1	160.05 (9)
C2—Fe1—C3—C4	119.36 (10)	Fe1—C1—C11—P1	−111.78 (9)
C8—Fe1—C3—C4	−118.54 (8)	C24—P1—C11—C12	−55.45 (8)
C1—Fe1—C3—C4	81.76 (7)	C18—P1—C11—C12	−165.79 (7)
C10—Fe1—C3—C2	−161.76 (15)	Pd1—P1—C11—C12	70.00 (8)
C9—Fe1—C3—C2	164.04 (8)	C24—P1—C11—C1	−179.46 (8)
C6—Fe1—C3—C2	45.79 (16)	C18—P1—C11—C1	70.20 (9)
C5—Fe1—C3—C2	−81.68 (7)	Pd1—P1—C11—C1	−54.01 (8)
C4—Fe1—C3—C2	−119.36 (10)	C1—C11—C12—C17	−111.01 (11)
C7—Fe1—C3—C2	79.78 (9)	P1—C11—C12—C17	123.19 (10)
C8—Fe1—C3—C2	122.09 (8)	C1—C11—C12—C13	67.35 (13)
C1—Fe1—C3—C2	−37.61 (7)	P1—C11—C12—C13	−58.45 (12)
C2—C3—C4—C5	−0.01 (14)	C17—C12—C13—C14	0.37 (16)
Fe1—C3—C4—C5	−59.26 (9)	C11—C12—C13—C14	−177.97 (10)
C2—C3—C4—Fe1	59.26 (8)	C17—C12—C13—P2	−178.78 (8)
C10—Fe1—C4—C3	163.73 (7)	C11—C12—C13—P2	2.87 (15)
C9—Fe1—C4—C3	121.91 (8)	C30—P2—C13—C14	85.14 (10)
C6—Fe1—C4—C3	−164.58 (13)	C36—P2—C13—C14	−26.70 (10)
C5—Fe1—C4—C3	−119.34 (10)	Pd1—P2—C13—C14	−140.51 (8)
C7—Fe1—C4—C3	47.93 (17)	C30—P2—C13—C12	−95.70 (10)
C2—Fe1—C4—C3	−37.70 (7)	C36—P2—C13—C12	152.46 (9)
C8—Fe1—C4—C3	80.22 (9)	Pd1—P2—C13—C12	38.64 (11)
C1—Fe1—C4—C3	−81.68 (7)	C12—C13—C14—C15	0.72 (17)
C10—Fe1—C4—C5	−76.93 (9)	P2—C13—C14—C15	179.91 (9)
C9—Fe1—C4—C5	−118.75 (8)	C13—C14—C15—C16	−1.47 (18)
C6—Fe1—C4—C5	−45.24 (17)	C14—C15—C16—C17	1.10 (18)
C3—Fe1—C4—C5	119.34 (10)	C15—C16—C17—C12	−0.02 (18)
C7—Fe1—C4—C5	167.27 (14)	C13—C12—C17—C16	−0.72 (17)
C2—Fe1—C4—C5	81.63 (7)	C11—C12—C17—C16	177.69 (11)
C8—Fe1—C4—C5	−160.44 (8)	C24—P1—C18—C19	130.75 (10)
C1—Fe1—C4—C5	37.65 (7)	C11—P1—C18—C19	−119.35 (10)
C3—C4—C5—C1	−0.10 (14)	Pd1—P1—C18—C19	4.22 (11)
Fe1—C4—C5—C1	−59.39 (8)	C24—P1—C18—C23	−50.03 (10)
C3—C4—C5—Fe1	59.29 (9)	C11—P1—C18—C23	59.86 (10)
C2—C1—C5—C4	0.17 (13)	Pd1—P1—C18—C23	−176.57 (8)
C11—C1—C5—C4	−177.98 (10)	C23—C18—C19—C20	0.80 (17)
Fe1—C1—C5—C4	59.22 (8)	P1—C18—C19—C20	180.00 (9)
C2—C1—C5—Fe1	−59.05 (8)	C18—C19—C20—C21	−0.91 (17)
C11—C1—C5—Fe1	122.80 (10)	C19—C20—C21—C22	−0.07 (18)
C10—Fe1—C5—C4	121.34 (8)	C20—C21—C22—C23	1.16 (19)
C9—Fe1—C5—C4	79.30 (9)	C21—C22—C23—C18	−1.27 (18)
C6—Fe1—C5—C4	162.63 (8)	C19—C18—C23—C22	0.28 (17)
C3—Fe1—C5—C4	−37.63 (7)	P1—C18—C23—C22	−178.95 (9)
C7—Fe1—C5—C4	−166.14 (15)	C18—P1—C24—C25	−97.61 (10)
C2—Fe1—C5—C4	−81.60 (8)	C11—P1—C24—C25	154.07 (9)
C8—Fe1—C5—C4	47.55 (17)	Pd1—P1—C24—C25	29.07 (11)
C1—Fe1—C5—C4	−119.57 (10)	C18—P1—C24—C29	81.20 (10)
C10—Fe1—C5—C1	−119.09 (7)	C11—P1—C24—C29	−27.12 (11)
C9—Fe1—C5—C1	−161.13 (7)	Pd1—P1—C24—C29	−152.12 (8)
C6—Fe1—C5—C1	−77.80 (8)	C29—C24—C25—C26	−1.65 (19)
C4—Fe1—C5—C1	119.57 (10)	P1—C24—C25—C26	177.21 (11)
C3—Fe1—C5—C1	81.94 (7)	C24—C25—C26—C27	1.2 (2)
C7—Fe1—C5—C1	−46.57 (18)	C25—C26—C27—C28	0.6 (2)
C2—Fe1—C5—C1	37.97 (6)	C26—C27—C28—C29	−1.9 (2)
C8—Fe1—C5—C1	167.12 (13)	C27—C28—C29—C24	1.44 (18)
C9—Fe1—C6—C10	37.91 (9)	C25—C24—C29—C28	0.33 (17)
C5—Fe1—C6—C10	−76.33 (9)	P1—C24—C29—C28	−178.47 (9)
C4—Fe1—C6—C10	−42.71 (18)	C36—P2—C30—C35	69.50 (11)
C3—Fe1—C6—C10	165.63 (13)	C13—P2—C30—C35	−38.11 (11)
C7—Fe1—C6—C10	119.45 (12)	Pd1—P2—C30—C35	−172.59 (8)
C2—Fe1—C6—C10	−160.61 (8)	C36—P2—C30—C31	−109.34 (10)
C8—Fe1—C6—C10	81.64 (10)	C13—P2—C30—C31	143.06 (9)
C1—Fe1—C6—C10	−118.44 (8)	Pd1—P2—C30—C31	8.57 (11)
C10—Fe1—C6—C7	−119.45 (12)	C35—C30—C31—C32	−1.05 (17)
C9—Fe1—C6—C7	−81.54 (10)	P2—C30—C31—C32	177.80 (9)
C5—Fe1—C6—C7	164.22 (8)	C30—C31—C32—C33	−1.24 (18)
C4—Fe1—C6—C7	−162.16 (13)	C31—C32—C33—C34	2.01 (19)
C3—Fe1—C6—C7	46.19 (17)	C32—C33—C34—C35	−0.47 (19)
C2—Fe1—C6—C7	79.94 (9)	C33—C34—C35—C30	−1.83 (18)
C8—Fe1—C6—C7	−37.81 (9)	C31—C30—C35—C34	2.58 (17)
C1—Fe1—C6—C7	122.11 (8)	P2—C30—C35—C34	−176.26 (9)
C10—C6—C7—C8	0.32 (15)	C30—P2—C36—C41	−5.45 (12)
Fe1—C6—C7—C8	59.45 (9)	C13—P2—C36—C41	105.01 (11)
C10—C6—C7—Fe1	−59.12 (9)	Pd1—P2—C36—C41	−131.55 (10)
C10—Fe1—C7—C6	37.54 (8)	C30—P2—C36—C37	176.27 (10)
C9—Fe1—C7—C6	81.59 (9)	C13—P2—C36—C37	−73.27 (11)
C5—Fe1—C7—C6	−41.8 (2)	Pd1—P2—C36—C37	50.17 (10)
C4—Fe1—C7—C6	163.12 (13)	C41—C36—C37—C38	−0.9 (2)
C3—Fe1—C7—C6	−161.73 (8)	P2—C36—C37—C38	177.46 (11)
C2—Fe1—C7—C6	−119.13 (8)	C36—C37—C38—C39	0.1 (2)
C8—Fe1—C7—C6	119.11 (13)	C37—C38—C39—C40	1.0 (2)
C1—Fe1—C7—C6	−76.70 (9)	C38—C39—C40—C41	−1.2 (2)
C10—Fe1—C7—C8	−81.57 (10)	C37—C36—C41—C40	0.70 (19)
C9—Fe1—C7—C8	−37.52 (9)	P2—C36—C41—C40	−177.56 (10)
C6—Fe1—C7—C8	−119.11 (13)	C39—C40—C41—C36	0.4 (2)
C5—Fe1—C7—C8	−160.89 (14)		

### Hydrogen-bond geometry (Å, °)

**Table:**

*D*—H···*A*	*D*—H	H···*A*	*D*···*A*	*D*—H···*A*
C6—H6···O1^i^	1.00	2.40	3.3365 (17)	156.
C15—H15···Cl1^ii^	0.95	2.70	3.4262 (12)	133.
C19—H19···Cl1	0.95	2.66	3.2410 (12)	120.
C23—H23···O1^i^	0.95	2.47	3.3239 (16)	149.
C25—H25···Cl1	0.95	2.70	3.5075 (13)	143.
C29—H29···O1^i^	0.95	2.45	3.3201 (16)	152.
C31—H31···Cl2	0.95	2.76	3.3893 (13)	125.
C42—H42A···O2	0.98	2.42	3.368 (2)	163.
C45—H45A···Cl1^iii^	0.98	2.83	3.6881 (16)	147.

Symmetry codes: (i) −*x*, −*y*+1, −*z*+1; (ii) *x*−1, *y*, *z*; (iii) −*x*+1, −*y*+1, −*z*+1.
